# Speak Up For Safety: Use of an Abbreviated Reporting Form to Increase Understanding of Near-miss and Adverse Events

**DOI:** 10.1097/pq9.0000000000000607

**Published:** 2022-10-03

**Authors:** Rebecca L. Kanaley, Tina Sosa, Kristen Brown

**Affiliations:** From the * Golisano Children’s Hospital Pediatric Nursing, University of Rochester Medical Center, Rochester, New York; † Golisano Children’s Hospital Pediatric Hospital Medicine, University of Rochester Medical Center, Rochester, New York.

## Introduction:

The reporting of adverse and near-miss safety events is an essential component of safety culture to understand potentially preventable harm and develop mitigation strategies. Identified barriers to reporting safety events include the burden of documentation, inadequate time, and lack of clarity around when, why, and how to report.^1,2^

## Objectives:

Through the iterative implementation of an abbreviated event reporting form, we aimed to increase event reporting by 10% over 24 months. Knowledge gained from these reports can inform cause analyses, debriefings, and improvement initiatives.

## Methods:

To increase the feasibility of reporting, we developed an optional abbreviated form, the SNAP form, for streamlined documentation. This form required seven fields, as compared to 16 on the original form. Plan-Do-Study Act cycles were utilized to refine the form and provide education. Interventions included a pilot period in the pediatric intensive care unit, an institution-wide announcement via leadership and unit safety nurses, sequential roll-outs to additional units, and incorporation of education into institution-wide “Error Prevention” training. The primary outcome measures were the rate of SNAP event reports filed per 1,000 patient-days and the total monthly event reports submitted. Metrics were tracked on run and statistical process control charts, utilizing established rules to detect special cause variation.

## Results:

**Fig. 1.**
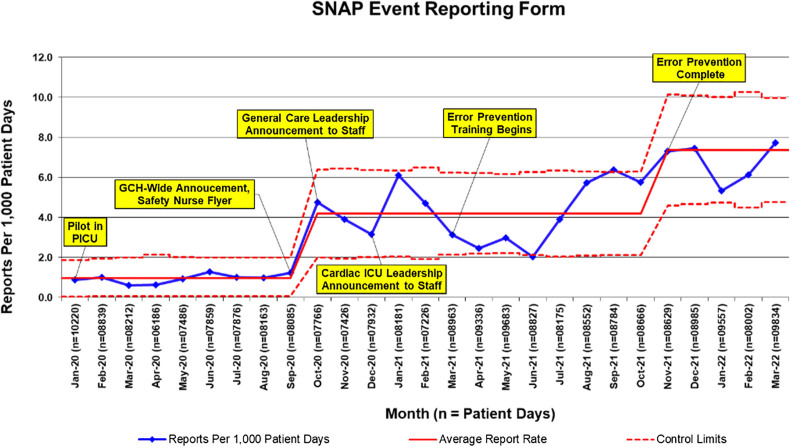
SNAP event reporting statistical process control U-chart.

**Fig. 2.**
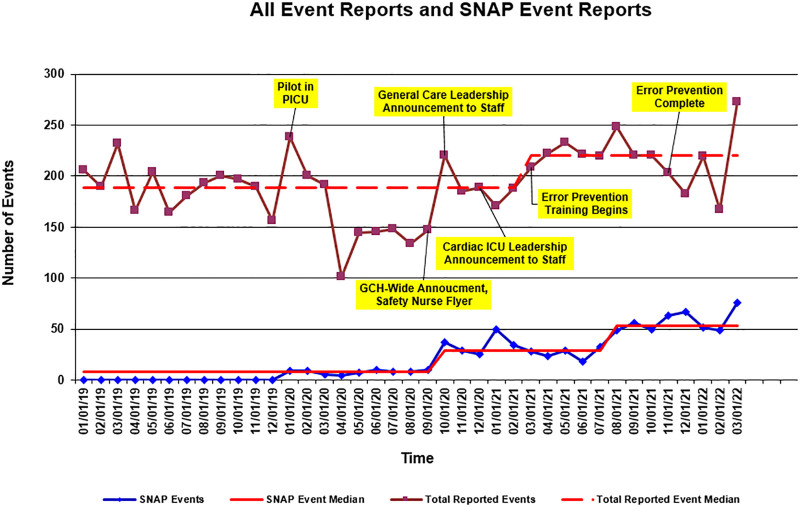
Overall event reporting run chart.

In association with our work, the average rate of SNAP forms filed per 1,000 patient-days increased from 0.96 to 7.38 over 24 months, with special cause variation observed on a U-chart (Fig. 1). The median overall number of events reported increased from 188 to 220 monthly (Fig. 2). The most common SNAP form event type was medication/fluid events, provision of care, and IV/vascular access events.

## Conclusions:

An abbreviated and feasible event reporting form was associated with a significant increase in reports. Incorporating relevant education into “Error Prevention” training was associated with the most notable increase. Knowledge generated can be utilized to develop strategies to reduce harm.

## References

[1] Evans SM Berry JG Smith BJ. Attitudes and barriers to incident reporting: a collaborative hospital study. Qual Saf Health Care. 2006;15:39–43. 16456208 10.1136/qshc.2004.012559 PMC2563993

[2] Rutledge DN Retrosi T Ostrowski G. Barriers to medication error reporting among hospital nurses. J Clin Nurs. 2018;27:1941–1949. 29495119 10.1111/jocn.14335

